# Quercetin Intervention Alleviates Offspring’s Oxidative Stress, Inflammation, and Tight Junction Damage in the Colon Induced by Maternal Fine Particulate Matter (PM_2.5_) Exposure through the Reduction of *Bacteroides*

**DOI:** 10.3390/nu12103095

**Published:** 2020-10-11

**Authors:** Wei Liu, Yalin Zhou, Yong Qin, Lanlan Yu, Ruijun Li, Yuhan Chen, Yajun Xu

**Affiliations:** 1 Department of Nutrition and Food Hygiene, School of Public Health, Peking University, Beijing 100083, China; liuwei19560912@163.com (W.L.); zhouyalin2017@163.com (Y.Z.); qinyong0520@163.com (Y.Q.); putaoeternal@163.com (L.Y.); aiesecarrow@163.com (R.L.); cyhan_ss@163.com (Y.C.); 2 Beijing Key Laboratory of Toxicological Research and Risk Assessment for Food Safety, Peking University, Beijing 100083, China

**Keywords:** air pollution, *Bacteroides*, intestinal barrier, offspring, quercetin

## Abstract

The influences of maternal fine particulate matter (PM_2.5_) exposure on intestinal oxidative stress, inflammation, tight junctions, and gut microbiota of offspring are not well understood. Moreover, research on the dietary intervention method has not been well studied. In our study, dams received PM_2.5_ and quercetin intervention during gestation and lactation, and then inflammation biomarkers, oxidative stress indicators, tight junction proteins, and gut microbiota in the colon of offspring were analyzed. Compared with the control group, lower catalase (CAT) and superoxide dismutase (SOD) activities, higher interleukin-17A (IL-17A) and interleukin-22 (IL-22), decreased ZO-1 and occludin expressions, and higher *Bacteroides* abundance were observed in the offspring mice of the PM_2.5_ group. However, higher CAT and SOD activities, lower IL-17A and IL-22 levels, increased ZO-1 and occludin expressions, and lower *Bacteroides* abundance were found in the quercetin groups. In addition, there was a negative correlation between *Bacteroides* abundance and CAT concentration. Additionally, *Bacteroides* abundance was positively related to IL-17A and IL-22 levels. These findings suggest that maternal PM_2.5_ exposure may have some certain effects on intestinal oxidative stress, inflammation, and tight junctions. Quercetin administration may protect the offspring against these adverse effects. Changes of *Bacteroides* abundance play an important role in the process.

## 1. Introduction

Epidemiological studies have reported that air pollution contributes to gastrointestinal diseases, such as inflammatory bowel disease [1,2]. These intestinal diseases may be related to the injury of the intestinal mucosal barrier, including oxidative stress, increased inflammation, and damaged tight junctions [3,4,5]. Fine particulate matter (PM_2.5_) could induce oxidative damage in tissue [6]. Oxidation stress affects the activity of antioxidant enzymes and the permeability and proliferation of epithelial cells; thus, oxidation stress is related to the barrier function of the gut. Vitro and vivo studies show PM_2.5_ has the ability to induce inflammation responses [7,8]. Occludin and ZO-1 are tight junction proteins, which help the cells of the intestinal epithelium adhere to each other and regulate permeability [9,10,11]. Bernard et al. reported that ZO-1 and occludin could be decreased by PM_2.5_ intratracheal instillation [12]. Mutlu et al. also found that particulate matter could decrease the epithelial barrier, which might be associated with rearrangement of the epithelial tight junction proteins [13].

Gut microbiota is involved in the maintenance of gut barrier function [14]. A growing body of evidence indicates that gut microbiota dysbiosis is implicated in defects in the gut barrier [15]. The environment has been identified as an important factor to influence gut microbiota. One study demonstrated that gut microbial diversity in the colon can be increased by PM_2.5_ exposure [16]. It is unknown whether the gut microbiota is related to oxidative stress, inflammation, and tight junctions induced by maternal PM_2.5_ exposure.

Quercetin, the most common flavonoid in food, is found in a number of plants such as apples, tea, strawberries, and onions [17,18]. According to clinical studies, the pharmacological effects of quercetin on inflammation [19] and oxidative stress have been confirmed. Moreover, quercetin can be transformed into other metabolites with the help of some microbes [20]. These metabolites have a number of biological activities, such as counteracting inflammation and oxidative stress [21,22].

Up to now, few studies have investigated the influences on intestinal oxidative stress, inflammation, tight junctions, and gut microbiota of offspring as their mothers were exposed to PM_2.5_ during the stages of pregnancy and lactation; moreover, there are no nutritional intervention methods. To explore these effects, dams were given PM_2.5_ and quercetin, and biomarkers of colon oxidative indicators, inflammation, tight junction proteins, and gut microbiota in the colon of offspring were analyzed.

## 2. Materials and Methods

### 2.1. PM_2.5_ and Quercetin

The dosages of PM_2.5_ and quercetin were determined in our previous research [23,24,25]. The PM_2.5_ sample was prepared by several processes: collecting, agitating, filtering, and lyophilizing. A detailed description can be seen in our previous articles [23,24]. Phosphate-buffered saline was used as the solvent to dissolve the PM_2.5_ powder. The control sample from unexposed filters was disposed equally. A solution of 0.15% carboxymethyl cellulose sodium was used to dissolve the quercetin (Sigma-Aldrich, Shanghai, China).

### 2.2. Animals and Treatment

Specific pathogen-free 8-week-old ICR mice (Beijing, China, SCXK2016-0010) were used in this study. After being quarantined for 7 days, female mice and male mice were put in the same cage overnight. If the vaginal plug was found the next morning, the day was defined as the gestational day (GD) 0. The day of natural production was defined as postnatal day (PND) 0. Every dam was caged alone. Forty dams were randomly divided into 5 groups, including the normal control (NC) group, the PM_2.5_ group (PM_2.5_ group), and 3 quercetin intervention groups (50, 100, and 200 mg/kg), with 8 dams in each subgroup. On PND 3, we kept 8 offspring in each litter, with 4 male mice and 4 female mice. On GD 3, 6, 9, 12, and 15, and PND 2, 5, 8, 11, 14, and 17, the dams were received intratracheal instillation under 3% isoflurane anesthesia. As shown in Table 1, the dams in the PM_2.5_ group and 3 quercetin intervention groups were exposed to 15 mg/kg PM_2.5_. The dams in the NC group received the suspension from extracts of a “blank” filter. The dams in the NC and PM_2.5_ groups were daily given 0.15% carboxymethyl cellulose sodium, while dams in the quercetin groups were daily given different doses of quercetin by gavage. Gavage was conducted every day from GD 0 to PND 21.

**Figure 1:**
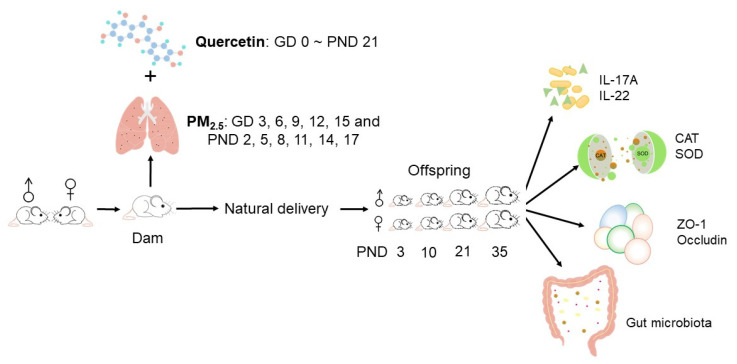
Experimental study design. A pair of male and female cubs born to the same mother were sacrificed on PND 3, 10, 21, and 35. Colonic microbiota, inflammatory biomarkers, antioxidases, and related proteins were detected. GD: gestational day; PND: postnatal day; IL: interleukin; CAT: catalase; SOD: superoxide dismutase.

On PND 3, 10, 21, and 35, one male and one female offspring born to the same mother were sacrificed. The use of mice was in compliance with the Peking University Guidelines for Animal Research (LA2016284). Figure 1 shows our study design.

### 2.3. Enzyme-Linked Immunosorbent Assays (ELISA) for Interleukin-17A (IL-17A) and Interleukin-22 (IL-22) in the Colon

Colonic tissue samples were collected on PND 3, 10, 21, and 35. As shown in Figure 1, IL-17A and IL-22 in the colon were measured using ELISA kits according to the kit instructions (Capitalbio, Beijing, China).

### 2.4. Biochemical Analysis for Catalase (CAT) and Superoxide Dismutase (SOD) in the Colon

Colorimetric assay kits (Nanjing Jiancheng, Jiangsu, China) were used to assay the activities of CAT and SOD in the colon (Figure 1). We conducted the process according to the kit instructions.

### 2.5. ZO-1 and Occludin in the Colon

ZO-1 and occludin in the colon were assessed by a Western blot. RIPA buffer (Thermal Scientific, USA) was used to homogenize the colon tissue. Then, the sample was centrifuged and prepared to measure the protein concentrations by BCA kits (P1511, Applygen Technologies Inc., Beijing, China). Each sample was loaded to the 8% sodium dodecyl sulfate-polyacrylamide gel electrophoresis and transferred to a polyvinylidene difluoride membrane. After being blocked for 4 h in a Tris-buffered saline/Tween 20, the membrane was incubated with primary antibodies of ZO-1 (1:1000; ab96587, Abcam), occludin (1:1000; ab216327, Abcam), and β-actin (1:5000; ab8227, Abcam) at 4 °C overnight. After that, the membrane was incubated with the secondary antibody. The immunoreactive protein bands were visualized using a Clarity Western ECL substrate kit (Sigma, CA, USA). Scion Image (Frederick, MD, USA) was used to perform the densitometry analysis of protein bands. The values were normalized against the intensity of β-actin (Figure 1).

### 2.6. Colonic Microbiota Analysis

Each colon content sample of offspring on PND 3, 10, 21, and 35 was used to analyze the gut microbiota (Figure 1). The details of 16S rDNA sequencing were introduced in our article [23]. Briefly, a QIAmp Fast DNA Stool Mini Kit (Qiagen, Germany) was used to extract the microbial genomic DNA in colon contents. Then, the V3-V4 region was amplified by PCR. Primers (5′-CCTACGGGRSGCAGCAG-3′ and 5′-GGACTACVVGGGTATCTAATC-3′) were marked with barcodes. After recovering the amplicons, a HiSeq platform (Illumina Inc., USA) was used to perform sequencing work. Sequencing data were measured using QIIME (version 1.9.1). Sequences with more than 97% identity were clustered into operational taxonomic units by Userach (version 7.0).

### 2.7. Statistical Analysis

Analysis of variance was used to analyze the effects on biomarkers of gut inflammation (IL-17A and IL-22), oxidative stress indicators (CAT and SOD), and proteins (ZO-1 and occludin). The least significant difference post-hoc test was used if equal variance existed, and Tamhane’s T2 post-hoc test was used if equal variance did not exist. Taxonomic relative abundances were calculated using the Kruskal–Wallis test, or the Mann Whitney U. Association between gut microbes and colonic inflammation or oxidative stress indicators was assessed by Spearman’s rank correlation analysis. *p* < 0.05 was regarded as statistically significant, and all tests were two-sided.

## 3. Results

### 3.1. Effects on Antioxidant Enzymes

**Figure 2:**
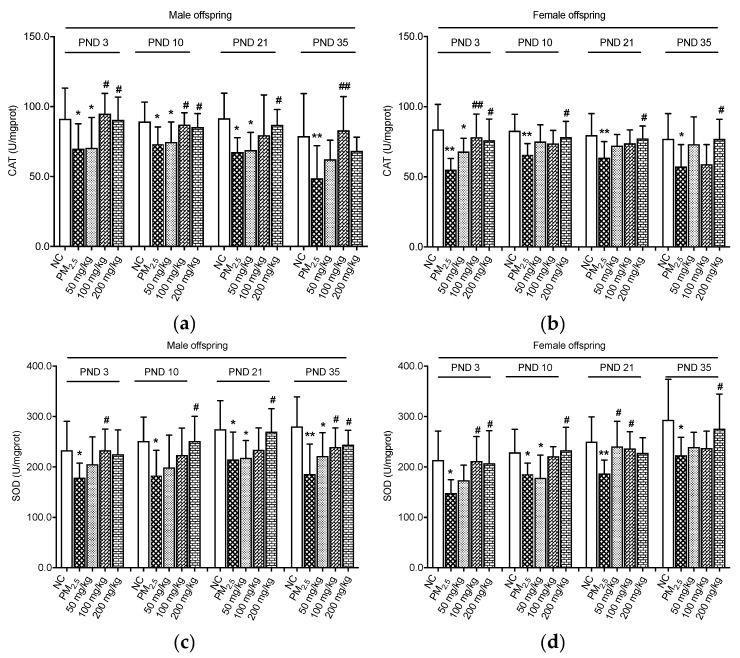
Effects on antioxidant enzymes of colon. (**a**) CAT levels of male offspring; (**b**) CAT levels of female offspring; (**c**) SOD levels of male offspring; (**d**) SOD levels of female offspring. Compared with the NC group, * indicates *p* < 0.05, ** indicates *p* < 0.01. Compared with the PM_2.5_ group, ^#^ indicates *p* < 0.05, ^##^ indicates *p* < 0.01. NC: normal control; PM_2.5_: fine particulate matter.

Activities of CAT and SOD of the offspring on PND 3, 10, 21, and 35 were assayed (Figure 2). It was evident that for male offspring, CAT levels in the PM_2.5_ groups were lower than those in the NC groups (*p* < 0.05, Figure 2a). On PND 3 and 10, the CAT levels were significantly elevated in the 100 and 200 mg/kg quercetin groups as compared with male samples in the PM_2.5_ group (*p* < 0.05). On PND 21, CAT concentrations of male offspring in the 200 mg/kg quercetin group were higher than those of the PM_2.5_ group (*p* < 0.05). The same effect was observed in male offspring in the 100 mg/kg quercetin group on PND 35 (*p* < 0.05). As shown in Figure 2b, the activity of CAT in the female offspring was significantly decreased in the PM_2.5_ groups compared with the NC groups (*p* < 0.05). Supplementation with quercetin at the doses of 100 and 200 mg/kg increased CAT activity compared with the PM_2.5_ group on PND 3 (*p* < 0.05). Moreover, 200 mg/kg quercetin administration significantly elevated the CAT of female offspring compared with the PM_2.5_ group on PND 10, 21, and 35 (*p* < 0.05).

The PM_2.5_ groups showed lower SOD levels of male offspring as compared with the NC groups (*p* < 0.05, Figure 2c). The middle dose of quercetin (100 mg/kg) could restore the SOD activity on PND 3 (*p* < 0.05). Quercetin 200 mg/kg significantly increased SOD concentrations of male offspring on PND 10 and 21 (*p* < 0.05). Additionally, mice treated with quercetin at doses of 100 and 200 mg/kg showed higher SOD concentrations on PND 35 than that of the PM_2.5_ group (*p* < 0.05). Maternal PM_2.5_ administration led to significant decreases in SOD activities of female offspring as compared with the control groups, as shown in Figure 2d (*p* < 0.05). Quercetin at two doses (100 and 200 mg/kg) showed protection effects against maternal PM_2.5_ exposure on PND 3 (*p* < 0.05). It was also observed that SOD concentrations of female offspring in the 200 mg/kg quercetin groups were higher than those of the PM_2.5_ groups on PND 10 and 35 (*p* < 0.05). A protective role of quercetin on SOD levels was also found in the 50 and 100 mg/kg quercetin groups on PND 21 (*p* < 0.05).

### 3.2. Effects on Inflammation Indicators

**Figure 3:**
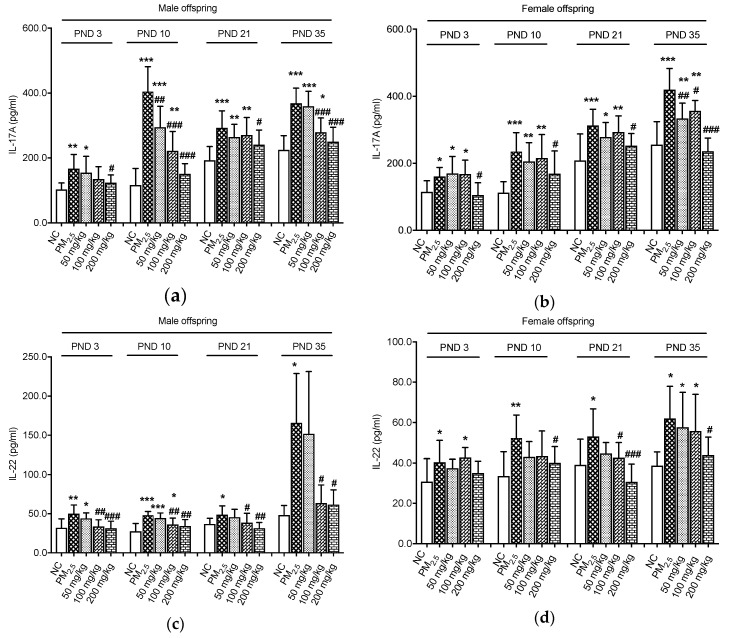
Effects on inflammation biomarkers of colon. (**a**) IL-17A levels of male offspring; (**b**) IL-17A levels of female offspring; (**c**) IL-22 levels of male offspring; (**d**) IL-22 levels of female offspring. Compared with the NC group, * indicates *p* < 0.05, ** indicates *p* < 0.01, *** indicates *p* < 0.001. Compared with the PM_2.5_ group, ^#^ indicates *p* < 0.05, ^##^ indicates *p* < 0.01, ^###^ indicates *p* < 0.001.

We further evaluated the levels of IL-17A and IL-22 in the colon (Figure 3). The results revealed that on PND 3, 10, 21, and 35, IL-17A concentrations of male offspring in the PM_2.5_ groups were higher than those in the NC groups (*p* < 0.05, Figure 3a). We also observed that the level of the anti-inflammatory cytokine IL-17A was down-regulated in the 200 mg/kg quercetin groups on PND 3 and 21, respectively, compared with the PM_2.5_ groups (*p* < 0.05). Furthermore, there were significant decreases of IL-17A of male offspring in the three quercetin groups on PND 10 compared with the PM_2.5_ group (*p* < 0.05). We found that the male offspring in the quercetin groups (100 and 200 mg/kg) exhibited a significant decrease of IL-17A levels as compared with the males in the PM_2.5_ group on PND 35 (*p* < 0.05). IL-17A of female offspring was significantly enhanced by maternal PM_2.5_ exposure (*p* < 0.05, Figure 3b). However, compared with the PM_2.5_ groups, lower IL-17A levels were observed in the 200 mg/kg quercetin groups on PND 3, 10, and 21 (*p* < 0.05). Similarly, female offspring in three quercetin groups showed striking reductions in the IL-17A expression on PND 35 compared with the control animals (*p* < 0.05).

As expected, male offspring in the PM_2.5_ groups showed higher expressions of IL-22 when compared with animals in the NC groups (*p* < 0.05, Figure 3c). However, the treatment with quercetin (100 and 200 mg/kg) provided marked decreases in IL-22 expression on PND 3, 10, 21, and 35 (*p* < 0.05). For female offspring, higher IL-22 levels were observed in the PM_2.5_ groups compared with the NC groups (*p* < 0.05, Figure 3d). A high dose of quercetin (200 mg/kg) could decrease the IL-22 concentration on PND 10 and 35 (*p* < 0.05). Likewise, on PND 21, IL-22 concentrations of female offspring in the 100 and 200 mg/kg quercetin groups were lower than those of the PM_2.5_ group (*p* < 0.05).

### 3.3. Effects on Colonic Proteins

**Figure 4:**
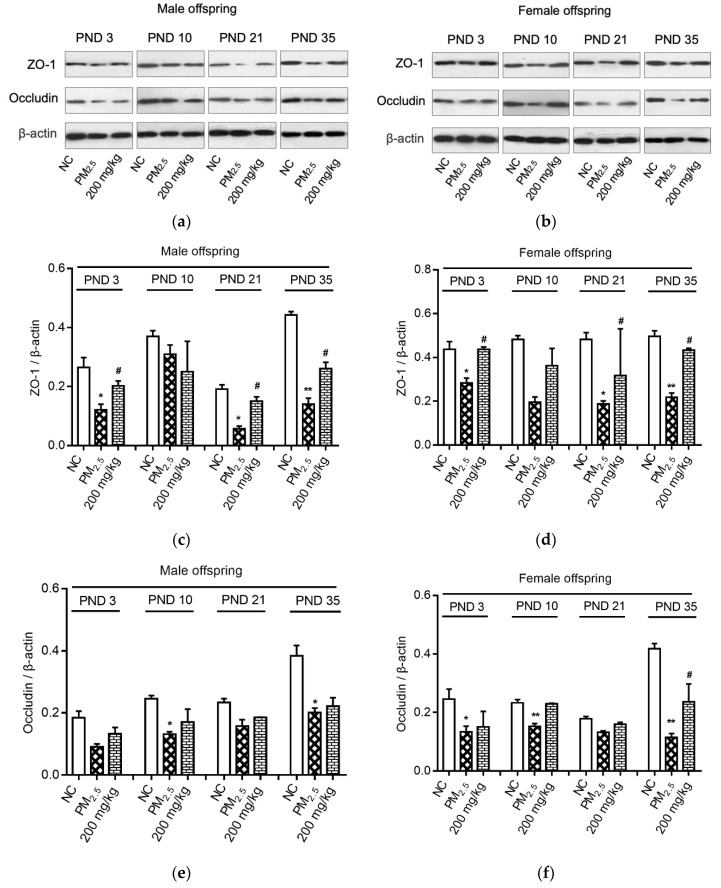
Effects on proteins of colon. (**a**) Immunoblots of ZO-1 and occludin expression of male offspring; (**b**) Immunoblots of ZO-1 and occludin expression of female offspring; (**c**) ZO-1 expressions of male offspring; (**d**) ZO-1 expressions of female offspring; (**e**) Occludin expressions of male offspring; (**f**) Occludin expressions of female offspring. Compared with the NC group, * indicates *p* < 0.05, ** indicates *p* < 0.01. Compared with the PM_2.5_ group, ^#^ indicates *p* < 0.05. The values were normalized against the intensity of β-actin, *n* = 3/group/gender/time.

Maternal PM_2.5_ exposure induced significant decreases in ZO-1 expressions in male and female offspring on PND 3, 21, and 35 (*p* < 0.05, Figure 4c,d). In contrast, the groups supplied with 200 mg/kg quercetin during PM_2.5_ exposure had significantly higher ZO-1 expression on PND 3, 21, and 35 (*p* < 0.05).

On PND 10 and 35, occludin expressions of male offspring were decreased by maternal PM_2.5_ exposure (*p* < 0.05, Figure 4e). However, quercetin supplementation had no significant effect on the occludin expression in male offspring (*p* > 0.05, Figure 4e). In addition, maternal PM_2.5_ exposure caused lower occludin expression in female offspring on PND 3, 10, and 35 (*p* < 0.05, Figure 4f). On PND 35, occludin expression in female offspring was reversed by quercetin supplementation (*p* < 0.05).

### 3.4. Axonomic Composition of Gut Microbiota

**Figure 5:**
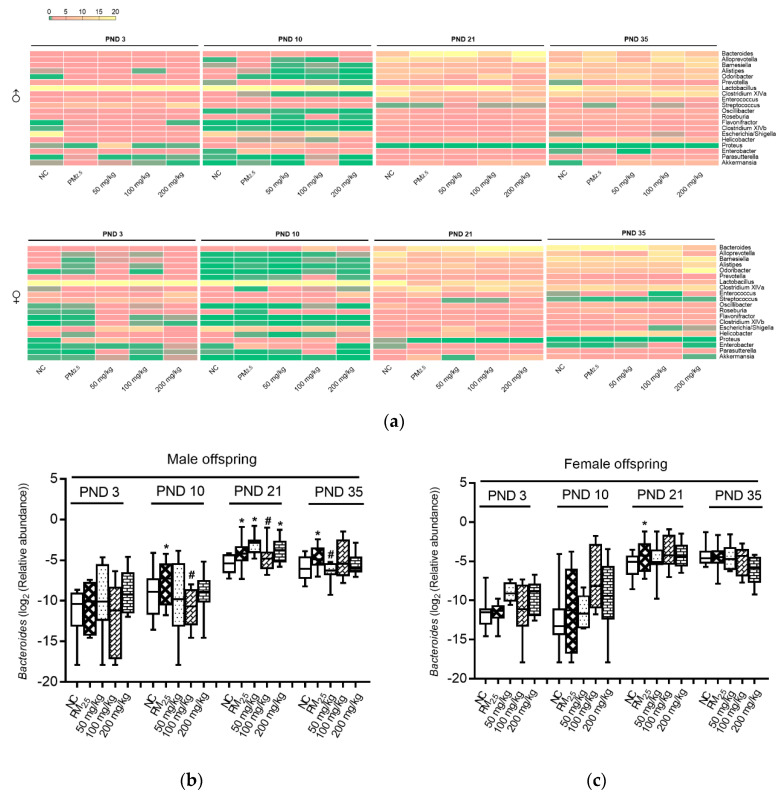
Axonomic composition of gut microbiota. (**a**) Axonomic composition of the top 20 genera; (**b**) *Bacteroides* of male offspring mice; (**c**) *Bacteroides* of female offspring mice. Compared with the NC group, * indicates *p* < 0.05. Compared with the PM_2.5_ group, ^#^ indicates *p* < 0.05.

Bacterial genera, including *Flavonifractor, Oscillibacter, Bacteroides, Alistipes, Proteus, Enterobacter,* and *Akkermansia* (which were found to be enhanced by PM_2.5_), were reversed by different doses of quercetin intervention (Figure 5a). In comparison with PM_2.5_ mice, quercetin intervention enhanced a variety of bacterial genera, including *Odoribacter, Streptococcus, Lactobacillus,* and *Roseburia* (Figure 5a; note that these species were initially reduced by PM_2.5_ compared with the NC group).

*Bacteroides* of male offspring mice on PND 10, 21, and 35, and female offspring mice on PND 21 in the PM_2.5_ groups were higher than those in the NC groups (Figure 5b,c). After quercetin intervention, *Bacteroides* of male offspring mice on PND 10, 21, and 35 in the quercetin groups were lower than those in the PM_2.5_ groups (Figure 5b).

### 3.5. Association between Bacteroides and Inflammation and Oxidative Indicators

**Figure 6:**
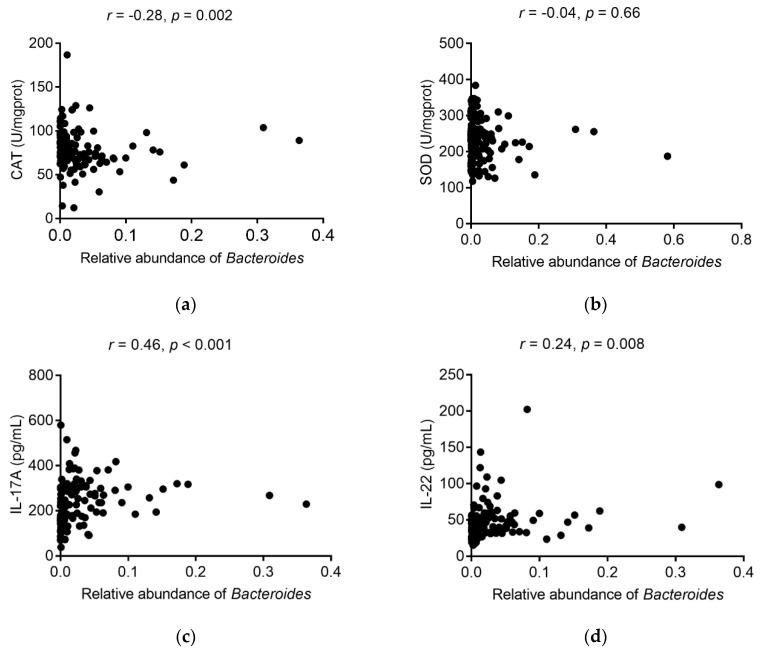
Correlation between *Bacteroides* and inflammation and oxidative indicators. (**a**) Correlation between *Bacteroides* and CAT; (**b**) Correlation between *Bacteroides* and SOD; (**c**) Correlation between *Bacteroides* and IL-17A; (**d**) Correlation between *Bacteroides* and IL-22. Spearman’s correlation analysis was used to analyze the relationship.

By Spearman’s correlation analysis (Figure 6), the result demonstrated that *Bacteroides* was negatively associated with colonic CAT concentration (*r* = −0.28, *p* = 0.002), whereas the *Bacteroides* abundance was positively related to IL-17A levels (*r* = 0.46, *p* < 0.001). Moreover, there was a positive correlation between *Bacteroides* and IL-22 (*r* = 0.24, *p* = 0.008). No clear correlation was found between the SOD concentration and the abundance of *Bacteroides* (*p* > 0.05).

## 4. Discussion

It is reported that PM_2.5_ is related to various health problems. However, there is no study to explore the effects on offspring‘s intestinal barriers when dams were exposed to PM_2.5._ Moreover, the intervention method has not been well illustrated. Our results indicated that maternal PM_2.5_ exposure led to crucial influences on intestinal oxidative stress, inflammation, and tight junctions of offspring mice, and quercetin administration may have some certain intervention effects. Gut microbiota may play an important role in this process.

Maternal PM_2.5_ exposure could affect gut barrier function. First of all, higher oxidative stress was found in the PM_2.5_ group, showing higher levels of CAT and SOD. Secondly, gut inflammation was observed in the PM_2.5_ group. IL-17A and IL-22 were both elevated. Thirdly, PM_2.5_ could have damaged epithelial tight junction proteins, showing low expression of occludin and ZO-1.

Gut barrier changes may be related to changes in gut microbiota. It can be seen that microbiota on PND 3 (colonization), 10 (breastfeeding), 21 (mixed feeding), and 35 (weaning) in the PM_2.5_ group were different from those in the control group. Polycyclic aromatic hydrocarbons [26], metals [27], and tetrachlorodibenzofuran [28] components were included in the PM_2.5_ [15], which can interact with intestinal flora. It is noteworthy that the relative *Bacteroides* abundance of male offspring mice increased in the PM_2.5_ group, and *Bacteroides* correlated with colonic CAT, IL-17A, and IL-22 levels. A study demonstrated that a higher level of *Bacteroides* was found in the colitis rats; moreover, their results also indicated that *Bacteroides* (*r* = 0.86, *p* = 0.001) was positively associated with the histological scores (including cell infiltration and tissue damage) [29]. Thus, higher *Bacteroides* caused by PM_2.5_ exposure was related to a damaged colon barrier. The above results indicated that PM_2.5_ might affect the intestinal barrier by acting on the intestinal flora.

Due to multiple reasons, it is impossible to solve PM_2.5_ pollution in a short period, so we hoped to reduce injury through diet intervention. Quercetin could improve the gut barrier function. Firstly, oxidative stress could be adjusted by quercetin. Increased levels of CAT and SOD were detected in quercetin groups significantly. Secondly, quercetin showed beneficial effects on inflammation changes. Levels of IL-17A and IL-22 of the quercetin groups were close to the NC groups. Thirdly, the expression of tight junction proteins was also improved. The expression of ZO-1 and occludin in the quercetin groups was higher than that in the PM_2.5_ group, indicating that quercetin could protect the colon mucosa barrier from damage.

We suspected that quercetin can improve the mucosa barrier by improving gut microbiota evolution of offspring. Gut microbiota abundance of offspring mice in quercetin intervention groups showed intervention effects. Taira et al. demonstrated that in rats, dietary polyphenols ameliorated imbalance in gut microbiota that was caused by a high-fat diet [30]. What is more, polyphenols could significantly inhibit the growth of *Bacteroidetes* and *Firmicutes*, and at the same time down-regulate the rate of *Bacteroidetes* to *Firmicutes* [31]. Another study demonstrated that the administration of quercetin was found to be effective in attenuating the *Firmicutes/Bacteroidetes* ratio [32]. In our study, we found that the *Bacteroides* that had been raised by PM_2.5_ decreased after the intervention of quercetin. Maternal PM_2.5_ exposure can cause oxidation and inflammation effects, moreover, the ZO-1 and occludin proteins also decreased. Quercetin may have protective effects on inflammation cytokines, antioxidases, and tight junction protein changes under PM_2.5_ exposure. These effects might be correlated with the changes in *Bacteroides*.

Although our study can provide new insights to learn about the influences of PM_2.5_, several limitations should be discussed. Firstly, our analysis only showed correlations between *Bacteroides* and indicators, but other associations may also exist with other unreported bacteria. Secondly, the way that gut microbiota influence barrier function has not been illuminated. Thirdly, the study is more about a hypothesis, and more studies will be needed to verify these opinions.

## 5. Conclusions

PM_2.5_ tracheal exposure during gestation and lactation could influence intestinal oxidative stress, inflammation, and tight junctions of offspring. Quercetin administration may have some certain intervention effects. Medium- and high-dose quercetin intervention showed better effects. Changes in *Bacteroides* play an important role in the process.

## Figures and Tables

**Table 1:** Animal treatment.

Group	N	PM_2.5_ (mg/kg)	Quercetin (mg/kg)
NC group	8	-	-
PM_2.5_ group	8	15	-
50 mg/kg quercetin group	8	15	50
100 mg/kg quercetin group	8	15	100
200 mg/kg quercetin group	8	15	200

Note: NC: normal control; PM_2.5_: fine particulate matter.
